# The Axolotl Fibula as a Model for the Induction of Regeneration across Large Segment Defects in Long Bones of the Extremities

**DOI:** 10.1371/journal.pone.0130819

**Published:** 2015-06-22

**Authors:** Xiaoping Chen, Fengyu Song, Deepali Jhamb, Jiliang Li, Marco C. Bottino, Mathew J. Palakal, David L. Stocum

**Affiliations:** 1 Department of Biology, School of Science, Indiana University-Purdue University Indianapolis, Indianapolis, Indiana, United States of America; 2 Department of Oral Biology, School of Dentistry, Indiana-University-Purdue University, Indianapolis, Indiana, United States of America; 3 Department of Restorative Dentistry, Division of Dental Biomaterials, School of Dentistry, Indiana-University-Purdue University Indianapolis, Indianapolis, Indiana, United States of America; 4 School of Informatics and Computing, Indiana University-Purdue University Indianapolis, Indianapolis, Indiana, United States of America; University of Dayton, UNITED STATES

## Abstract

We tested the ability of the axolotl (*Ambystoma mexicanum*) fibula to regenerate across segment defects of different size in the absence of intervention or after implant of a unique 8-braid pig small intestine submucosa (SIS) scaffold, with or without incorporated growth factor combinations or tissue protein extract. Fractures and defects of 10% and 20% of the total limb length regenerated well without any intervention, but 40% and 50% defects failed to regenerate after either simple removal of bone or implanting SIS scaffold alone. By contrast, scaffold soaked in the growth factor combination BMP-4/HGF or in protein extract of intact limb tissue promoted partial or extensive induction of cartilage and bone across 50% segment defects in 30%-33% of cases. These results show that BMP-4/HGF and intact tissue protein extract can promote the events required to induce cartilage and bone formation across a segment defect larger than critical size and that the long bones of axolotl limbs are an inexpensive model to screen soluble factors and natural and synthetic scaffolds for their efficacy in stimulating this process.

## Introduction

Mammalian bones fail to regenerate across gaps that exceed the critical size defect (CSD), defined as the smallest defect that cannot be bridged by regenerated skeletal tissue over the life of the animal [1]. CSDs in human patients are most often caused by the surgical necessity of removing a bone segment damaged by trauma or disease [2]. For example, penetrating and blast injuries are particularly damaging to the extremities, creating a challenge for repairing a CSD to avoid amputation [3]. Several surgical methods are used to repair CSDs, including bone grafts and prosthetics, but the functional result is often unsatisfactory. Bone autografts, while immunologically acceptable, are not ideal because of a limited number of donor sites and potential donor morbidity. Processed (decellularized) bone allografts circumvent this limitation, but are prone to complications such as infection, non-union, and stress fracture [4, 5].

Orthopedic regenerative medicine seeks new strategies aimed at regeneration across CSDs in both intramembranous and endochondral bones. These strategies have included implanting osteoinductive and osteoconductive scaffolds, with or without osteogenic cells and/or growth factors or growth factor genes [2, 6–11]. While partly successful, none of these strategies has attained clinical status due to less than optimal bone regeneration and/or integration with the remaining bone ends. Suboptimal bone regeneration in CSDs of endochondral long bones may be due in part to a focus on direct bone regeneration, rather than on reproducing the process of endochondral bone development that takes place during fetal development and fracture repair, which is to first develop a cartilage template that is subsequently replaced by bone [10, 12]. Since hypertrophied chondrocytes release factors that induce osteogenesis [13], regeneration of cartilage theoretically should be sufficient to lead to osteogenesis.

Most studies on segment defects in endochondral bones have been carried out on mice, rats, rabbits and sheep. Amphibians have been used extensively for studies on the regeneration of amputated limbs, and to a lesser extent for experiments on regeneration of individual limb skeletal elements in unamputated limbs [10, 14]. Extirpated skeletal elements were reported able to regenerate in the unamputated limbs of young urodele larvae, but not adults [15]. Removal of bones from adult newt limbs followed by amputation through the middle of the defect resulted in regeneration of the skeletal parts distal to the amputation plane by a regeneration blastema, but the skeletal elements proximal to the amputation plane were not regenerated [16–18]. Feng et al [19] demonstrated a failure to bridge large segment defects made in one of the two tarsal bones of the unamputated adult *Xenopus* hind limb.

**Fig 1:**
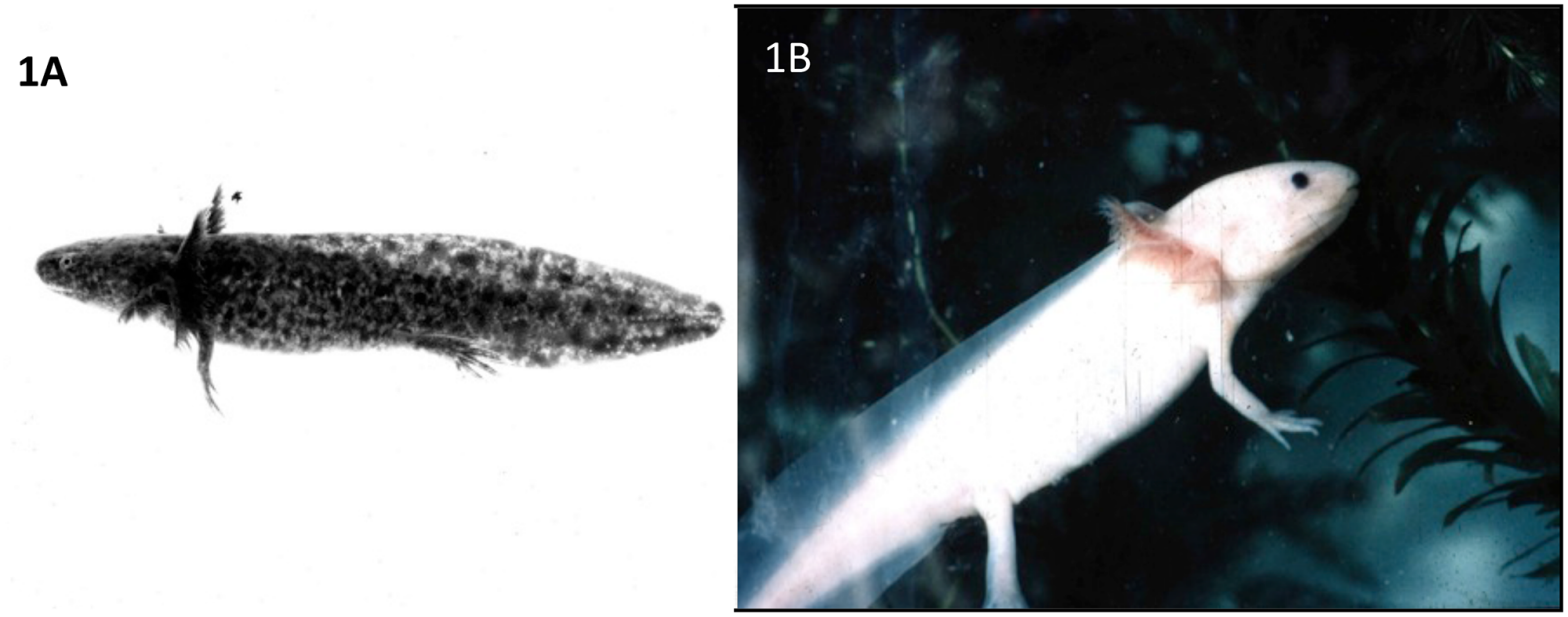
The young adult axolotl, *Ambystoma mexicaum*. Left, wild-type animal with dark skin pigmentation. Right, the white mutant, which lacks skin melanophores.

The urodele salamander *Ambystoma mexicanum* (axolotl) (Fig 1) has been used for over a century to study the regeneration of amputated limbs [20]. Axolotls, like *Xenopus* and mammals, are unable to regenerate across large defects in long bones [21, 22]. Axolotls have several advantages for the study of segment defect regeneration, including ease and low cost of maintenance in the laboratory, rapid wound healing, no requirement for bone fixation, ease of post-operative care, as well as low morbidity and mortality. For these reasons, we began to systematically develop the axolotl fibula as an experimental model to investigate the basic biology of segment defect repair and to screen combinations of soluble factors, gene constructs, natural, and synthetic scaffolds for their efficacy in promoting regeneration across CSDs of long bones. As a first step, we report here the results of experiments on regeneration across segment defects in the fibula of young adult axolotls. We found that the fibula could regenerate to repair fractures and across defects of 10% and 20% of its length in the absence of any therapeutic intervention, but could not regenerate across defects of 40% or 50%. In a screen of growth factor combinations and protein extracts of axolotl whole limb and regeneration blastema tissues, we found that a combination of BMP-4 and HGF, as well as limb tissue protein extract, but not blastema extract of amputated limbs, stimulated skeletal regeneration across 50% defects when delivered by a pig small intestine submucosa (SIS) scaffold.

## Materials and Methods

### In Silico identification of growth factors involved in fracture repair

Multiple bone morphogenetic proteins (BMPs) have been implicated in skeletal development and regeneration [23]. We have previously used bait proteins and bioinformatics techniques to mine the literature and construct pathways and networks of protein interactions that operate during blastema formation in amputated axolotl limbs [24, 25]. Here we employed the same techniques to mine the literature on fracture repair, cartilage regeneration, and bone regeneration to identify growth factors in addition to BMPs that might be used to stimulate regeneration across segmental defects. Keywords related to the process of cartilage differentiation were identified and submitted to the in-house literature-mining tool BioMAP. BioMAP uses a multi-level approach to identify these entities: (1) part-of speech (POS) tagging by Brill Tagger to identify the noun phrases from the text; (2) biological entity classification (such as genes, proteins, cell type, organism etc.) for the noun phrases by using the UMLS and other dictionaries such as LocusLink; and (3) Hidden Markov Models and N-gram machine-learning methods, to identify biological entities not discovered by dictionary matching.

The information extracted by BioMAP was normalized using the protein and gene names from the UniProt database. The Human Protein Reference Database (HPRD) was then used to identify growth factors and transcription factors from this gene/protein list. These growth factors and transcription factors were used to determine the predominant pathways and networks of protein interaction in cartilage regeneration, using MetaCore (GeneGO Inc). These were further analyzed using four topological parameters of the CytoHubba plugin [26] in Cytoscape (http://www.cytoscape.org/) to select the proteins most commonly identified as significant. The topological properties evaluated were: bottleneck nodes, maximal cliques (MCC), eccentricity, and maximum connected component (MNC).

Eleven growth factors emerged from this analysis: These were FGF-2, PDGF-A, PDGF-B, PDGF-D, EGF, HGF, TGF-β2, TGF-β3, Follistatin, VEGF-A, and Lefty-2. Nine of these factors have been implicated experimentally in fracture repair, nine in bone regeneration, and seven in cartilage regeneration (Table 1). TGF-β2 and FGF-2 have been implicated in soft callus chondrogenesis, and HGF and PDGF-BB accelerate fracture repair [27]. HGF does this by facilitating the expression of BMP receptors [28]. Lefty-2, which is involved in left/right asymmetry during embryogenesis [29], has not been implicated experimentally in skeletal development and regeneration.

**Table 1:** Growth factors implicated by bioinformatics analysis in cartilage and bone regeneration, and in fracture repair.

Growth Factor	Fracture Repair	Cartilage Regeneration	Bone Regeneration
FGF-2	+	+	+
PDGF-A	+	+	+
HGF	+	+	+
TGF-β 1, 2	+	+	+
TGF-β3	+	+	+
Lefty-2	+	+	+
EGF	+	-	+
PDGF B	+	-	+
PDGF D	+	-	+
VEGF A	-	+	+
Follistatin	+	-	-

### Preparation of growth factor and protein extract solutions

Seven of these growth factors, in addition to BMP-4, were commercially available: VEGF-A, HGF, FGF-2, TGF-β3, PDGF-AA, PDGF-BB, and EGF. We eliminated PDGF-BB from the experiments since the bioinformatics data indicated that it plays no role in cartilage regeneration, but kept PDGF-AA because the data indicate that it does play a role. The growth factors had sequence alignment to their *Xenopus* counterparts (the animal closest to the axolotl for these purposes) of 55% (EGF) to 99% (BMP-4). Five different combinations of BMP-4 and the other selected growth factors were tested for their ability to promote regeneration across a 50% segment defect (Table 2). The concentrations chosen for the growth factors were estimated from published reports in which single growth factors were used to promote regeneration across segmental defects in rodent bones [30–34]. All growth factors were purchased from PeproTech (Rocky Hill, NJ) and stock solutions of each prepared according to instructions provided by the company. Catalogue numbers for the human growth factors used were: BMP-4, AF-120-05ET; PDGF-AA, AF-100-13A; TGF-β3, AF-100-36E; FGF-2, AF-100-18B; EGF, AF-100-18B. Catalogue numbers for the murine growth factors were: VEGF, AF-450-32; HGF, 315–23. The stock solutions were used to prepare solutions of the combinations, which were then diluted to the final treatment concentration with filtered 0.8X amphibian phospho-buffered saline (aPBS). The composition of this saline was 0.11M NaCl, 0.002M KCl, and 0.01M phosphate buffer, pH 7.4.

**Table 2:** Growth factor combinations and concentrations tested for their ability to promote cartilage regeneration across 50% defects in the axolotl fibula.

Treatment Group		GF Concentration
**1**	Amphibian PBS	—
**2**	BMP-4	10 ng/μl
	VEGF	25 ng/μl
**3**	BMP-4	10 ng/μl
	HGF	10 ng/μl
**4**	BMP-4	10 ng/μl
	VEGF	25 ng/μl
	HGF	10 ng/μl
**5**	BMP-4	10 ng/μl
	VEGF	25 ng/μl
	HGF	10 ng/μl
	FGF-2	8 ng/μl
**6**	BMP-4	10 ng/μl
	VEGF	25 ng/μl
	HGF	10 ng/μl
	FGF-2	8 ng/μl
	EGF	10 ng/μl
	TGFβ-3	2ng/μl
	PDGF-AA	10 ng/μl
**7**	Blastema protein extract	7μg/μl
**8**	Limb protein extract	6μg/μl

We also tested the ability of protein extract from axolotl medium bud regeneration blastema and from intact whole limb tissue to promote regeneration (Table 2). Freshly collected tissues were ground in liquid nitrogen and cell lysis buffer in the presence of proteinase inhibitors (RayBio, Norcross, GA). Bradford assay indicated that the protein concentration of the extracts was approximately 5.76 mg/ml. The concentrations of blastemal protein extract and whole limb protein extract used were 7 μg/ml and 6 μg/ml, respectively. These extracts induced no inflammatory reaction when injected into the muscle of the axolotl hind limb.

### Animals and Surgery

We used both wild-type (dark) and white (dd mutant) axolotls obtained as small larvae from the Ambystoma Stock Center at the University of Kentucky and raised to a length of 12–15 cm (nose to tail tip, young adults). Both types are widely used in limb regeneration studies and are not known to differ in any respect related to regeneration. They were maintained in individual containers with daily changes of artificial pond water (10% Holtfreter solution) and fed salmon chow daily (Rangen, Buhl, Idaho). Axolotls (unfed for 24 hrs prior to surgery) were anesthetized by immersion in 0.1% (w/v) of MS-222 (Fisher Scientific, Pittsburgh PA) in 10% Holtfreter solution buffered with bicarbonate to pH 7.3. The age of the animals used was approximately the same, but because of individual differences in individual growth rates, the limbs were not uniform in size from animal to animal.

A simple cut with microscissors through the mid-diaphysis was used to create a fracture of the fibula. To create a segment defect, the length of the fibula was first measured externally with calipers. A longitudinal cut was made with microscissors along the posterior aspect of the hind limb zeugopodium and the muscle pushed to one side to expose the fibula. A length of bone equivalent to a defect of 10%, 20%, 40% or 50% of the total fibula length was then excised from the middle of the bone. The average length of the fibula was 7 mm. The average length of the defects was thus approximately 0.7 mm, 1.4 mm, 2.8 mm, and 3.5 mm, respectively. Wounds were closed with two sutures of #6 silk thread (Fine Science Tools Inc., Foster City, CA). The animals were kept immersed in 0.01% MS-222 to control pain during a recovery period of two hours and then returned to artificial pond water. The wounds healed within one week without other treatment. Control limbs (no intervention or implant of scaffold alone) were harvested at intervals of one to six months, and fixed for one week in 10% formalin.

**All animal care and experiments were carried out in strict accordance with the recommendations in the Guide for the Care and Use of laboratory animals of the National Institutes of Health. The protocol was approved by the School of Science Institutional Animal Care and Use Committee of Indiana University-Purdue University Indianapolis (Protocol SC 226R).**

Defects of 50% were used to test growth factor combinations and protein extracts. SIS scaffolds loaded with growth factor combinations, blastema extract, or tissue extract were inserted into 50% defects immediately after removal of the bone segment from the fibula and the wounds closed with two #6 silk sutures. Controls consisted of limbs in which the fibular defect received no treatment or was implanted with scaffold soaked overnight at 4°C in 0.8x aPBS. Growth factor-treated and tissue extract-treated limbs were harvested at intervals of two to six months, and fixed for one week in 10% formalin.

### Loading of SIS Scaffold with Growth Factors or Tissue Extracts

An eight-strand biodegradable braid of SIS, a generous gift from Cook Biotech (West Lafayette, IN), was used to deliver combinations of growth factors, blastema extract, and tissue extract to 50% segment defects. The braid was half the diameter of the fibula and hydrated to the diameter of the fibula when soaked in growth factor or extract solution prior to implantation into a segment defect. The braid was cut into pieces approximating the length of the segment defect that were then sterilized for 30 min with UV light. For each set of implants, five sterile pieces were immersed together overnight at 4°C in 50 μl of sterile growth factor or tissue extract solution contained in the inverted cap of a 1 ml Eppendorf tube. The cap in turn was placed in a small culture dish floored with sterile moist paper to maintain humidity and prevent changes in concentration of the solutions.

### Release Kinetics of BMP-4 from SIS Scaffold

Kinetics of protein release was analyzed for BMP-4. Three scaffolds at a time were soaked in 50 μl of BMP-4 solution (10 ng/μl) at 4°C overnight. Each scaffold was then serially transferred to new Eppendorf tubes containing 50 μl of 0.8x aPBS and allowed to release for 2 hr, 4hr, 8hr, 1 day, 2 days, and 3 days at room temperature. The concentration of BMP-4 per 50 μl of BMP-4 released into the 50 μl of aPBS at each time point was measured using a human BMP-4 Quantikine ELISA kit (R & D Systems, Minneapolis, MN).

### X-Ray and Computed Tomography

All of the fixed limbs for each time point were first imaged by X-ray (PIXARRAY 100, Bioptics, San Jose, Costa Rica) and then by microcomputed tomography (micro-CT), using a high-resolution desktop imaging system (SkyScan 1172, Allentown, PA). The micro-CT scans included the region from the distal end of the femur to the proximal tarsus and were obtained using an X-ray source set at 60kV and 167 μA over an angular range of 180° (rotational steps of 0.9°) with a 17-μm pixel size. NRecon Reconstruction software was used to construct cross-sectional images with up to 8000 x 8000 pixels, which were then used to reconstruct a volumetric 3-D image. These techniques image ossified tissue, but do not detect cartilage formation. Bone volume per total volume (BV/TV) was calculated at the defective region in each animal.

### Histology

After X-ray and micro-CT imaging, half of the samples for each time point were stained in 0.25% methylene blue for cartilage matrix, or double-stained in methylene blue and alizarin red for both cartilage and bone matrix. The other half of the fixed specimens was decalcified in Calciclear (Fisher Scientific, Pittsburgh, PA) for three weeks at room temperature, embedded in paraffin (Fisher Scientific), sectioned at 10 μm, and stained with Ehrlich’s hematoxylin and eosin (Fisher Scientific). Some slides were stained with Gomori’s Trichrome stain (Polysciences, Warrington, PA) after being re-fixed overnight in Bouin’s solution after the re-hydration step. Imaging was done on a Leica model DM2000 light microscope equipped with a camera (Leica Microsystems, Buffalo Grove, IL).

### Fluorochrome Imaging

Two fluorochromes were used to measure early bone regeneration in untreated 10% vs. 50% defects. On the day of surgery, a 1% calcein solution (Sigma Chemical, St. Louis, MO) was injected subcutaneously into the proximal one-third of the hind limb zeugopodium at a dose of 15μg/gm body wt. This fluorochrome fluoresces green. One week after the surgery, a solution of 3% alizarin complexone red was injected at the same site at a dose of 30 μg/gm body wt. This fluorochrome fluoresces red. The limbs were harvested at 3 weeks post-surgery and fixed in 10% neutral formalin solution for one week. The soft tissues were removed with jeweler’s forceps and the fibular bone on either side of the defect was imaged whole with Leica fluorescent optics for red and green fluorescence (Leica Microsystems, Buffalo Grove, IL). Two color images were merged using Image J software and the extent of bone regeneration was assessed by measuring the length of red color extending from green color on the anterior, middle and posterior sides of the bone.

## Results

### Controls: Untreated Defects and Defects Implanted With Scaffold Alone

Table 3 summarizes the results of regeneration in the absence of any treatment or after implanting 8-braid SIS alone. One hundred percent of untreated fractures showed regenerated skeletal tissue by two months post-operation and 80%-88% of the 10% and 20% segment defects, respectively, had repaired the defect with regenerated cartilage and bone by three months post-operation. The failure to regenerate in two of the 10% defects and one 20% defect may have been due to factors such as individual variation in response to injury, misalignment of bone ends, osteoclastic erosion of the cut ends of the fibula to increase the defect size, or some combination of these.

**Table 3:** Regeneration of cartilage in the absence of intervention across defects of 10%, 20%, 40% and 50% in the axolotl fibula.

Defect Type	Regeneration (# of Limbs)
Fracture	12/12
10%	8/10
20%	7/8
40%	0/8
50%	0/10

Fracture data is two months post-operation; 10%, 20%, 40% and 50% data is three months post-operation.

**Fig 2:**
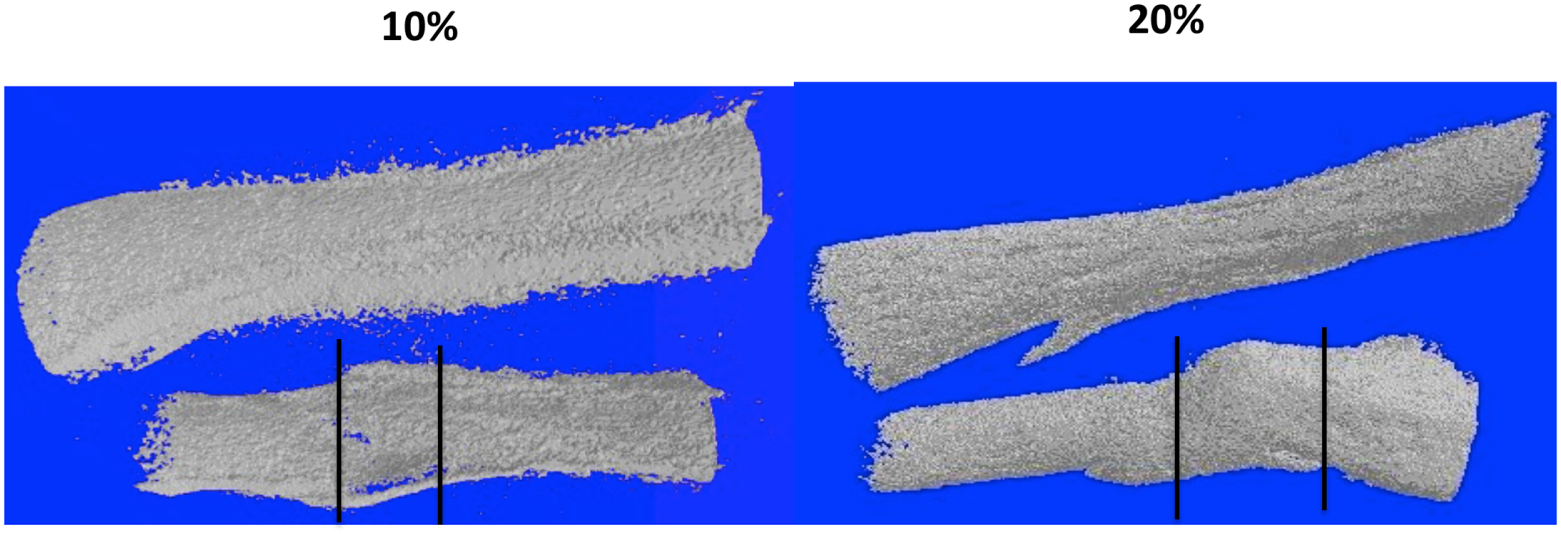
10% and 20% defects imaged by micro-CT at two months post-operation. The tibia is to the top and the fibula at the bottom. Lines indicate the boundaries of the regenerated bone. Note that these boundaries represent the bulk of the regenerated bone, which extends beyond the defect region *per se*.

**Fig 3:**
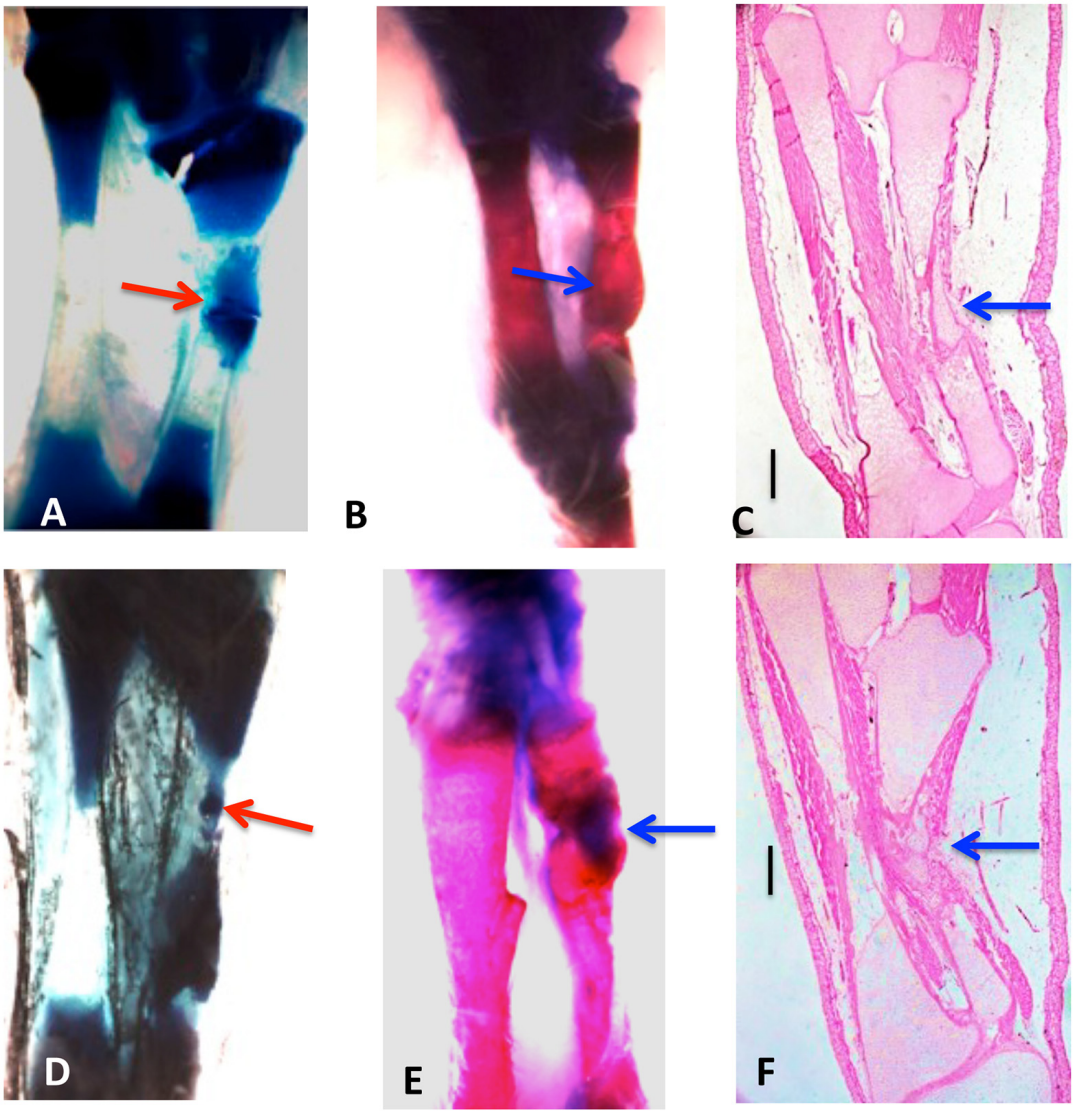
Whole mounts of control 10% (A, B, C) and 20% defects (D, E, F) stained with methylene blue (A, D) or methylene blue/alizarin red (B, E), and H & E-stained sections(C, F), three months post-operation. Tibia is on the left and fibula on the right in all photos. Both cartilage and bone (arrows) have regenerated across the defects. Bars in (C, D) equal 400 μm.

X-ray imaging proved to be of little use, but micro-CT scans showed that ossification had taken place in the defects (Fig 2). Fig 3 illustrates examples of MB/AR staining of whole mounts and H&E-stained sections of regenerated tissue at 3 months post-operation. The new bone formed an irregular bridge connecting the cut ends of the fibula.

The process of regeneration appeared to be very similar in fractures and 10% segment defects, which were bridged within 2–3 weeks with cartilage that ossified earlier than in 20% defects. Examination of sections at three weeks post-operation indicated that in the 20% defects, the ossification centers arose not in the center of the cartilage bridge, as in the fetal development of endochondral bones, but in the cartilage regenerating from each end of the fibula, suggesting that regeneration took place from both ends of the cut bone toward the center. Flattened chondrocytes, hypertrophying chondrocytes, and regions of osteogenesis with osteoblasts and osteoclasts were observed extending from the ends of the bone in these sections.

**Fig 4:**
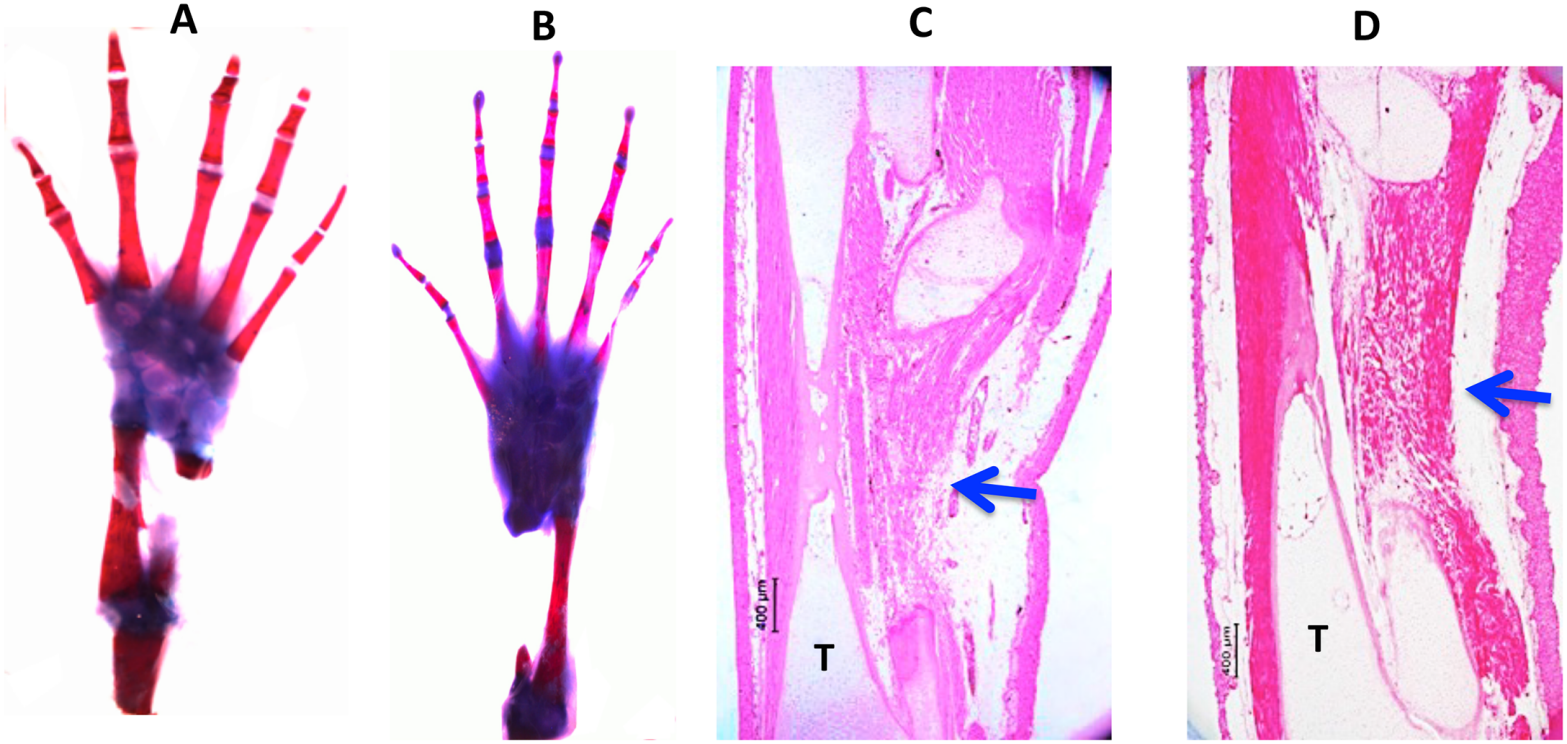
Control 40% (A, C) and 50% (B, D) defects three months post-operation. (A, B), whole mounts stained with methylene blue/alizarin red. C, D, H & E-stained sections. No regeneration has taken place. In the sections, muscle and connective tissue have filled the defect space (arrows). T = tibia. Bars = 400 μm.

None of the untreated 40% and 50% defects showed any regeneration by three months post-operation, or even at 5–6 months. X-ray and CT scans were negative at all time points. MB/AR staining and sections stained with H&E showed clearly that at three months post-operation the cut ends of the fibula had capped off without further regeneration and that the defects were filled with disorganized fibrous connective tissue and regenerated muscle **(** Fig 4). Using a definition of CSD as the smallest size defect that did not regenerate over the course of our experiments (up to six months), these data indicate that the CSD for the axolotl fibula is greater than 20%, but less than 40%.

**Fig 5:**
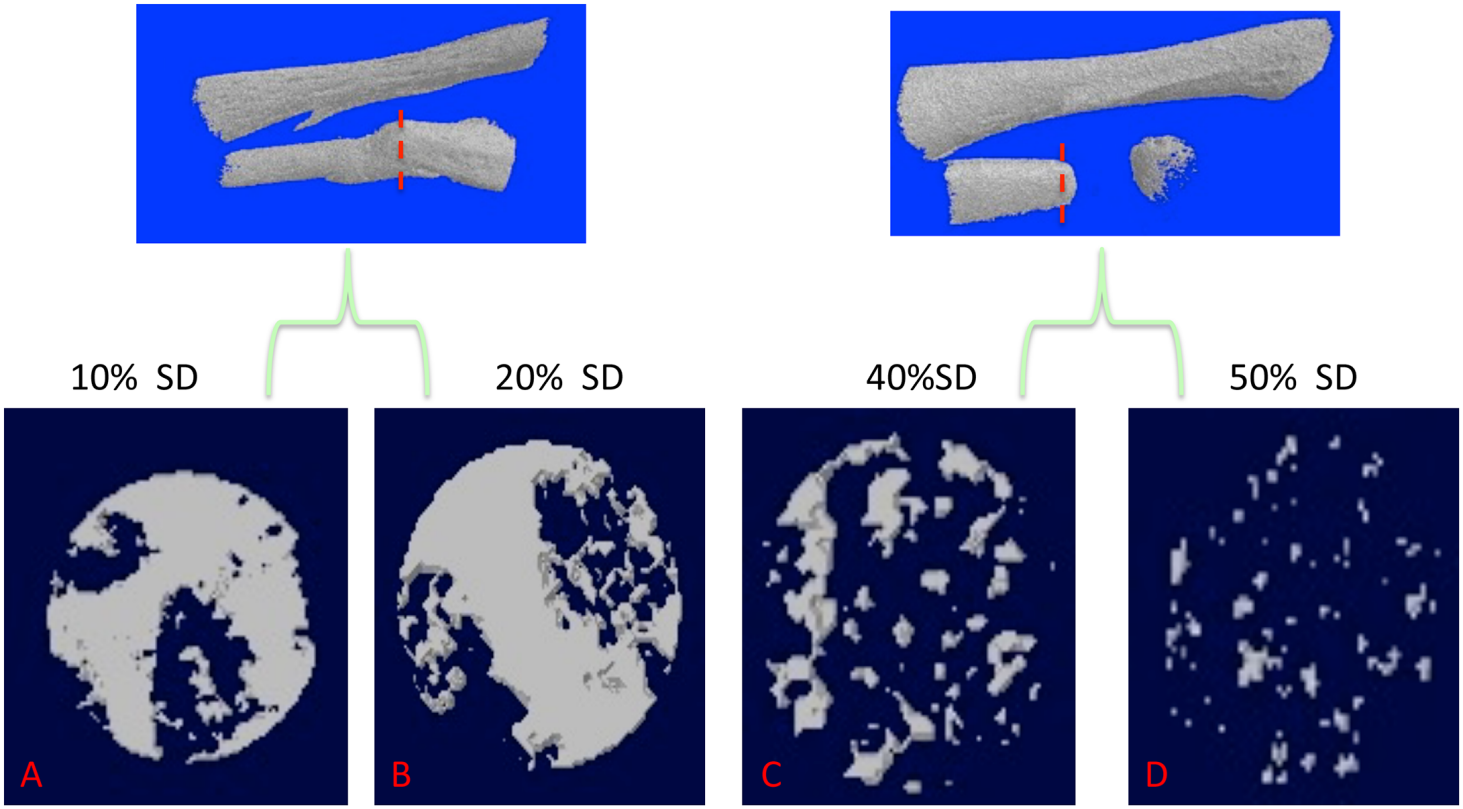
Bone volume fraction of regenerated bone in 10%- 50% defects at two months post-operation. The 20% defect is the same one illustrated in Fig 2. Top, red dashed lines show the cross-section level in the center of the 10% and 20% defects (A, B) and the 40% and 50% defects (C, D) where the measurements were taken. Both 10% and 20% defects regenerated a high volume fraction of bone, whereas there was much less regeneration in the 40% defects, and minimal regeneration in the 50% defects, which only regenerated at the cut ends of the fibula.

**Fig 6:**
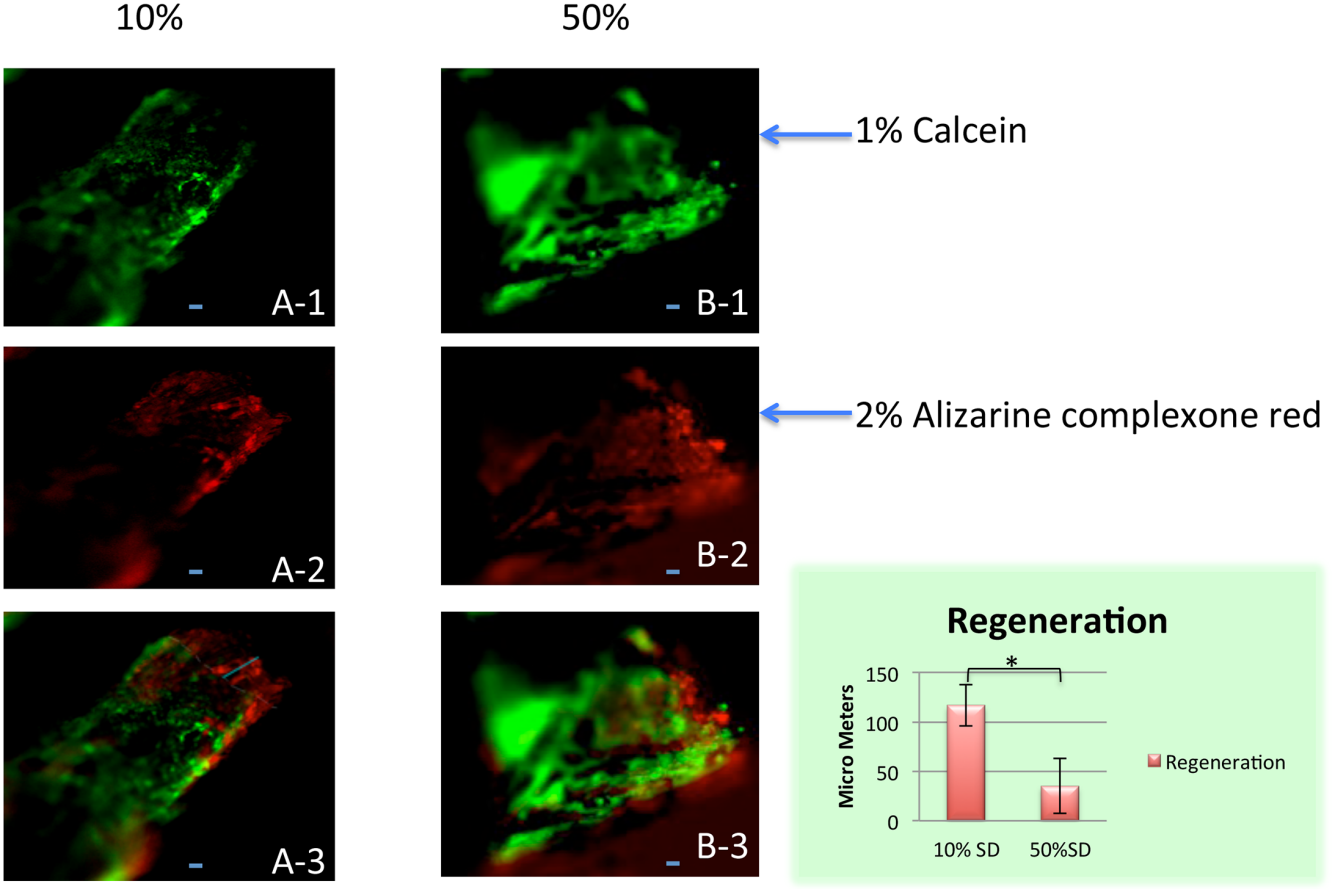
Use of two fluorochromes to measure bone regeneration in 10% and 50% segment defects after 3 weeks of regeneration. 1% calcein was injected on the day of surgery at a dose of 15ug/mg body weight. 2% alizarine complexone red was injected 1 week post-surgery at a dose of 30ug/mg body weight. Scale bar = 50 μM. Samples (six for each group) were harvested 3 weeks post-surgery. (A-1, A-2, A-3) is a 10% defect; (B-1,B-2,B-3) is a 50% defect. (A-1, B-1), calcein green fluorescence; (A2, B2), alizarine complexone red fluorescence; (A-3, B-3), red and green color merged. In the merged image of the 10% defect (A-3), the red color extends beyond the green, indicating that regeneration is taking place. By contrast, red and green show no separation in the merged image of the 50% defect (B-3), indicating lack of regeneration. The extent of regeneration was obtained by measuring the length of red extending beyond green on the anterior, middle and posterior points of the anterior-posterior axis of the fibula and averaging the three measurements (bar graph). The difference between the 10% and 20% defects was significant at p = 0.05 (Unpaired T-test). Scale bar = 50 μm.

Fig 5 shows the newly regenerated bone volume fraction (BVF) in the middle of the regenerated region where bone density is expected to be least. In the 10% defects, the BVF was 74.4% and in the 20% defects it was 70.6%. The BVF for the 40% defects was 38.7%, and for the 50% defects, 11.3%. We used calcium-binding fluorochromes [35] to visualize sites of active mineralization in 10% vs. 50% defects in the fibula (Fig 6). In merged images of calcien (green) and alizarin complexone (red), regeneration of bone is clearly underway in the 10% defect by 3 weeks post-operation, as indicated by the separation of the green and red colors, whereas virtually no color separation had taken place at either end of the 50% gap.

**Fig 7:**
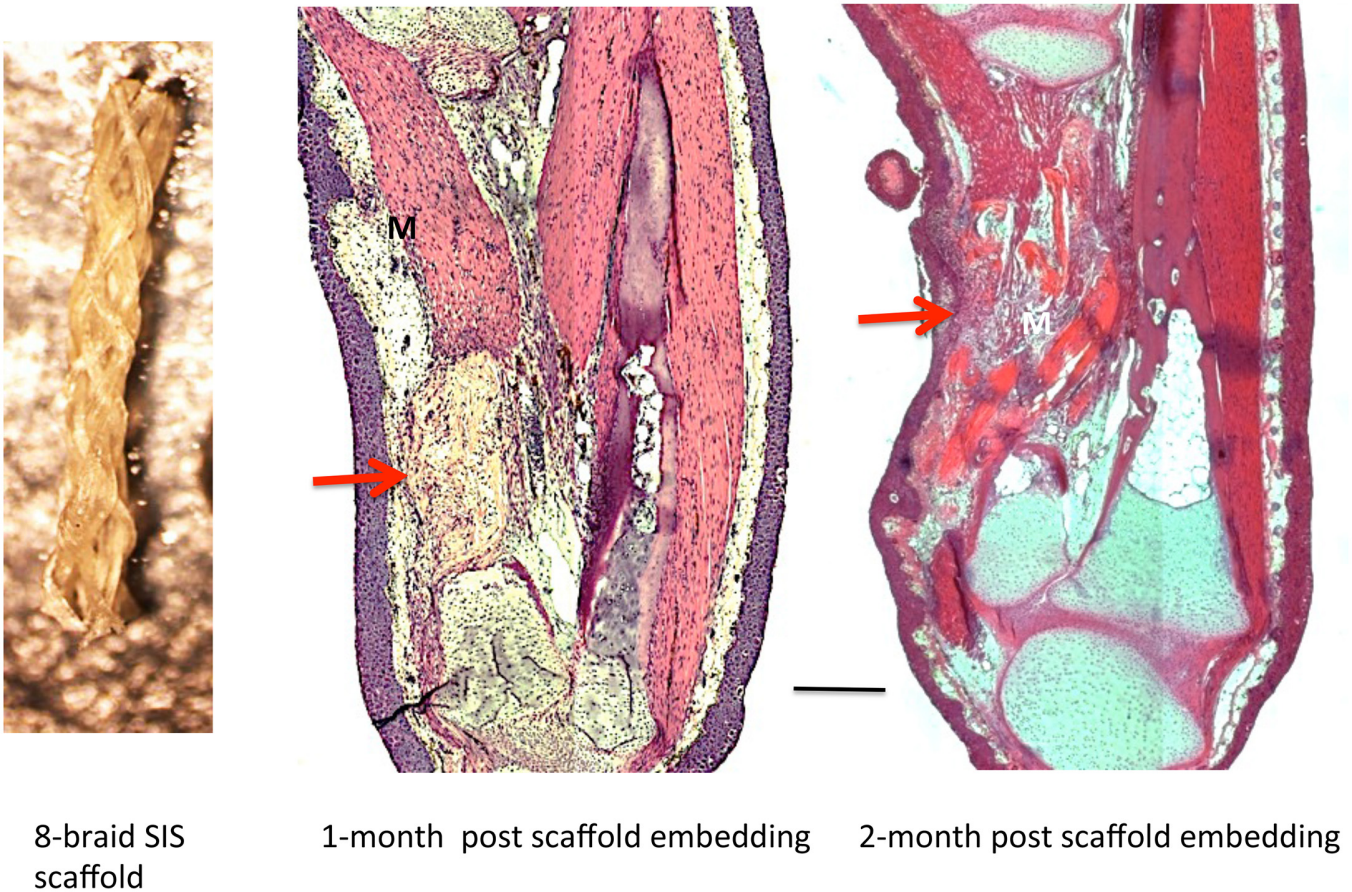
Result of implanting 8-braid SIS scaffold soaked in 0.8 aPBS. Left, 8-braid scaffold prior to hydration. Middle and right, 50% defect at one and two months, respectively, after embedding SIS scaffold alone. No cartilage has regenerated. In the one-month specimen, the implanted scaffold is still visible within the gap (arrow). At two months, the scaffold is largely degraded, and connective tissue and muscle has regenerated into the gap (arrow). M = muscle. Scale bar = 400 μm.

To determine whether the 8-braid SIS scaffold would by itself promote regeneration across 40% and 50% defects, we implanted four scaffolds into 40% defects and 16 scaffolds into 50% defects. The braid was hydrated by dipping it into the growth factor solvent (sterile 0.8x aPBS) prior to insertion into the defect. Trichrome and H&E staining of sections were used to monitor degradation of the scaffold and the tissue filling the defect space. After three months there was no visible skeletal regeneration in any of the defects and they were filled with disorganized connective tissue and regenerated muscle (Fig 7). These results demonstrate that the SIS scaffold was unable by itself to stimulate regeneration of skeletal tissue.

### Effect of Growth Factors and Tissue Extracts on 50% Defects

Fifty percent defects were implanted with 8-braid SIS soaked in growth factor, blastema extract or tissue extract solutions. This defect size was chosen to provide a regenerative challenge well beyond the CSD. We noted that upon examination of whole mounts and sections that the bone at the cut ends of the fibula in many cases had undergone substantial regression, making the segment defect closer to 70%.

**Fig 8:**
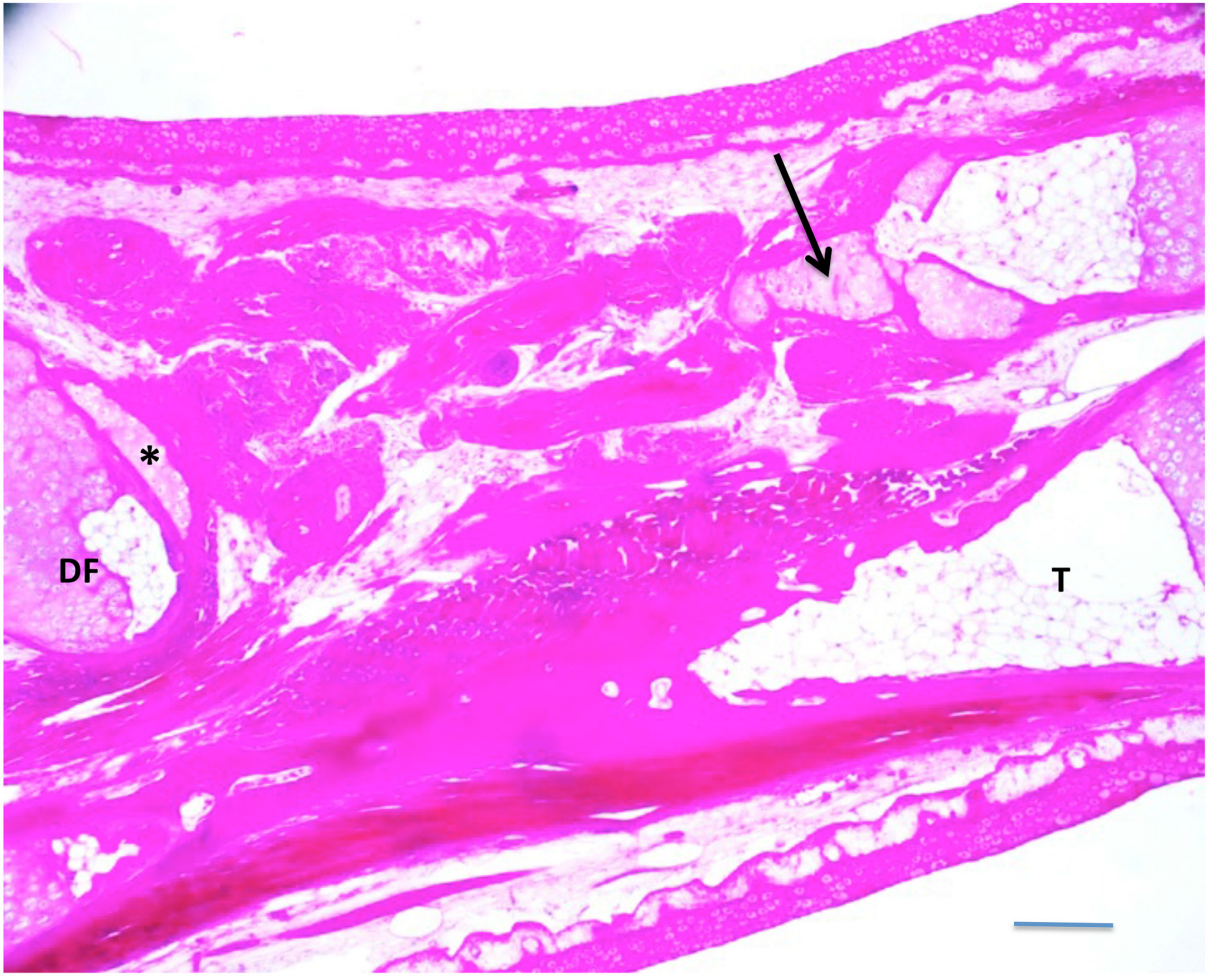
A 50% defect treated with BMP4/VEGF/HGF/EGF/FGF2/PDGF-AA/TGFβ-3, three months post-implant. A small amount of cartilage (arrow) regenerated from the proximal end of the fibula. The distal end of the fibula (DF) was severely angled with respect to the proximal end. A small amount of cartilage appears to have regenerated transversely on the distal fibula stump (asterisk). T = tibia. Scale bar = 400 μm.

**Fig 9:**
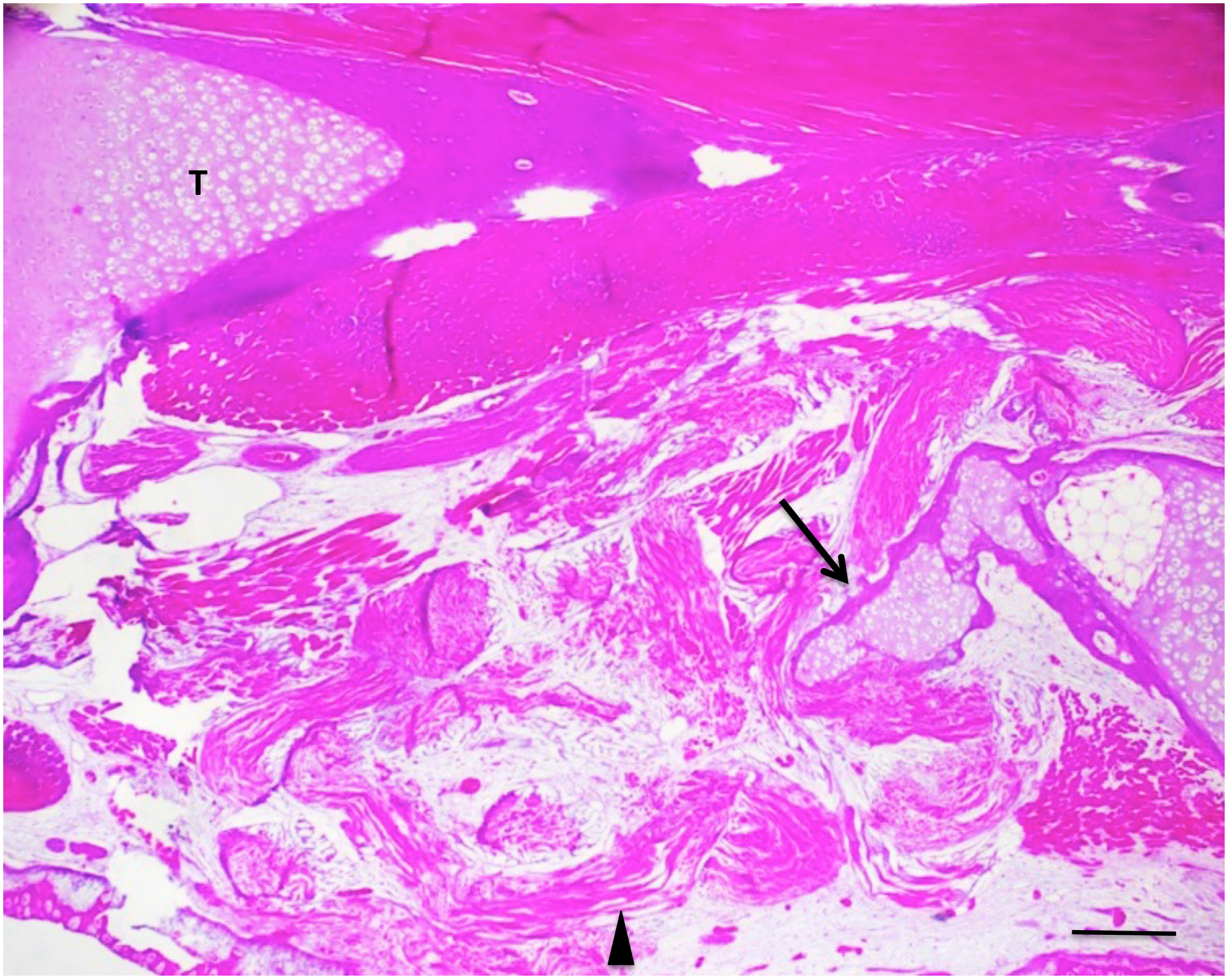
A 50% defect treated with BMP4/VEGF, three months post-operation. An irregular tongue of cartilage with periosteal bone collar (arrow) has regenerated from the distal end of the fibula. Remnants of the SIS scaffold can be seen within the defect, along with muscle and connective tissue (arrowhead). T = tibia. Scale bar = 400 μm.

Table 4 shows the results. The degree of regeneration fell into two categories, partial and extensive. Partial regeneration was defined as bridging less than 25% of the defect, whereas extensive regeneration was defined as bridging 50% or more of the defect. The 7-factor combination yielded one case of partial regeneration out of 20 limbs (Fig 8), and the BMP-4/VEGF combination yielded two cases of partial regeneration out of 20 limbs (Fig 9). The regenerated skeletal tissue consisted of irregular tongues of cartilage. No cases of extensive regeneration were observed.

**Table 4:** Number of limbs in which growth factors and limb tissue extract induced partial or extensive regeneration across 50% defects in the axolotl fibula at three months post-operation.

	Partial Regeneration	Extensive Regeneration	Total	
Seven Factors	1/20	0/20	1/20	(5%)
BMP-4/VEGF	2/20	0/20	2/20	(10%)
BMP-4/HGF	2/20	4/20	6/20	(30%)
Limb Tissue Extract	2/18	4/18	6/18	(33.3%)
Other GF combinations	0/20	0/20	0/20	(0.0%)
Control (scaffold alone)	0/16	0/16	0/16	(0.0%)

Growth factor combinations and blastema extract were tested on a total of 20 limbs each (both hindlimbs of 10 animals), and tissue extract on a total of 18 limbs (both hindlimbs of 10 animals; one animal died). Partial regeneration means that a small amount of cartilage/bone regenerated from one or both cut ends of the fibula. Extensive regeneration means that skeletal regeneration spanned more than 50% of the defect.

**Fig 10:**
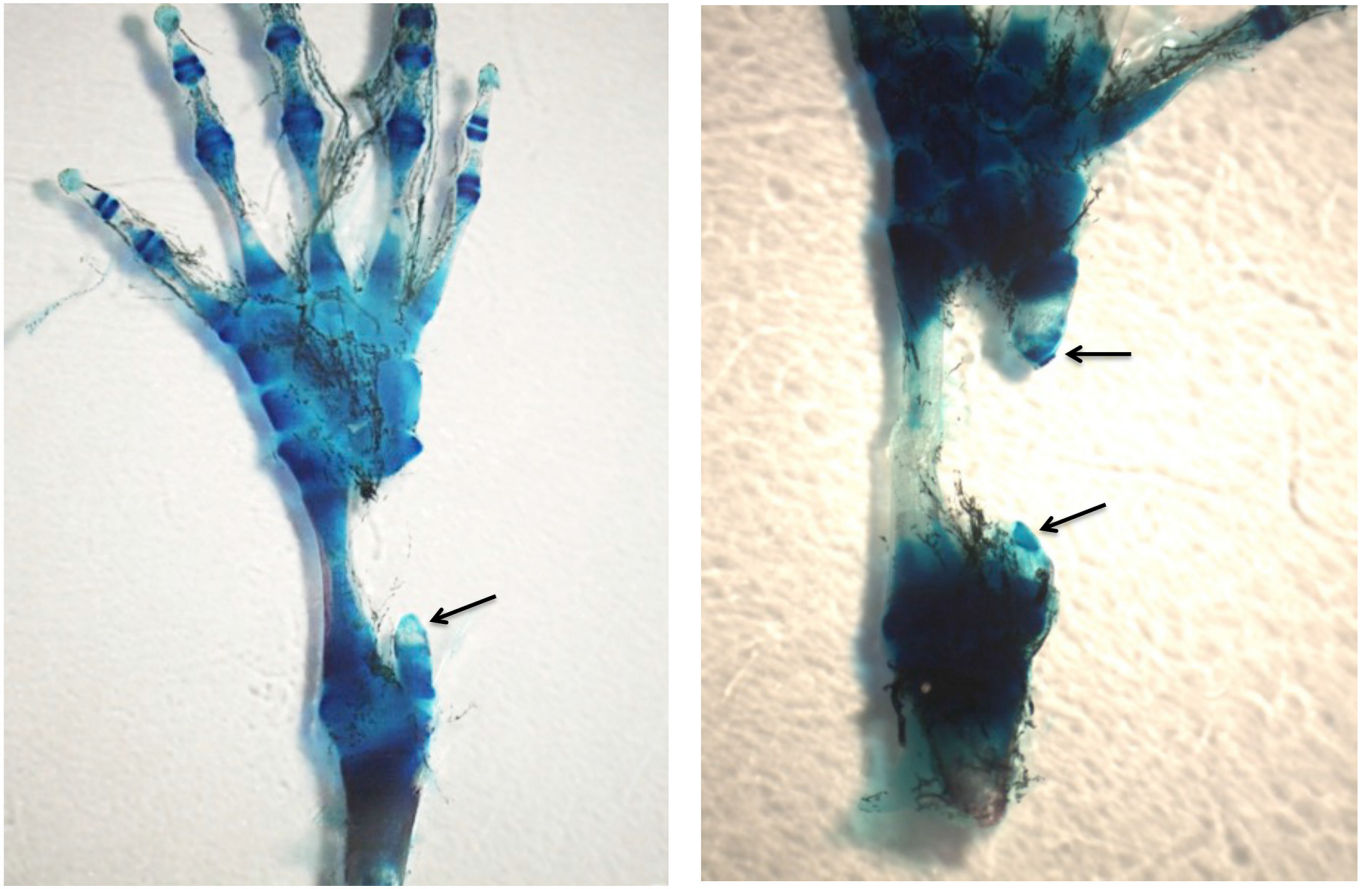
Left, MB-stained whole mount of 50% defect treated with BMP4/HGF, 3 months post-operation. A short cone of cartilage (arrow) regenerated from the proximal end of the fibula. No regeneration took place from the distal end. Right, MB-stained whole mount of 50% defect treated with BMP4/HGF in which short cones of cartilage regenerated from both the proximal and distal ends of the fibula (arrows). Both specimens illustrate a common phenomenon encountered after removal of 50% of the bone, namely that the remaining distal and/or proximal bone segments regress to create closer to a 70% gap.

**Fig 11:**
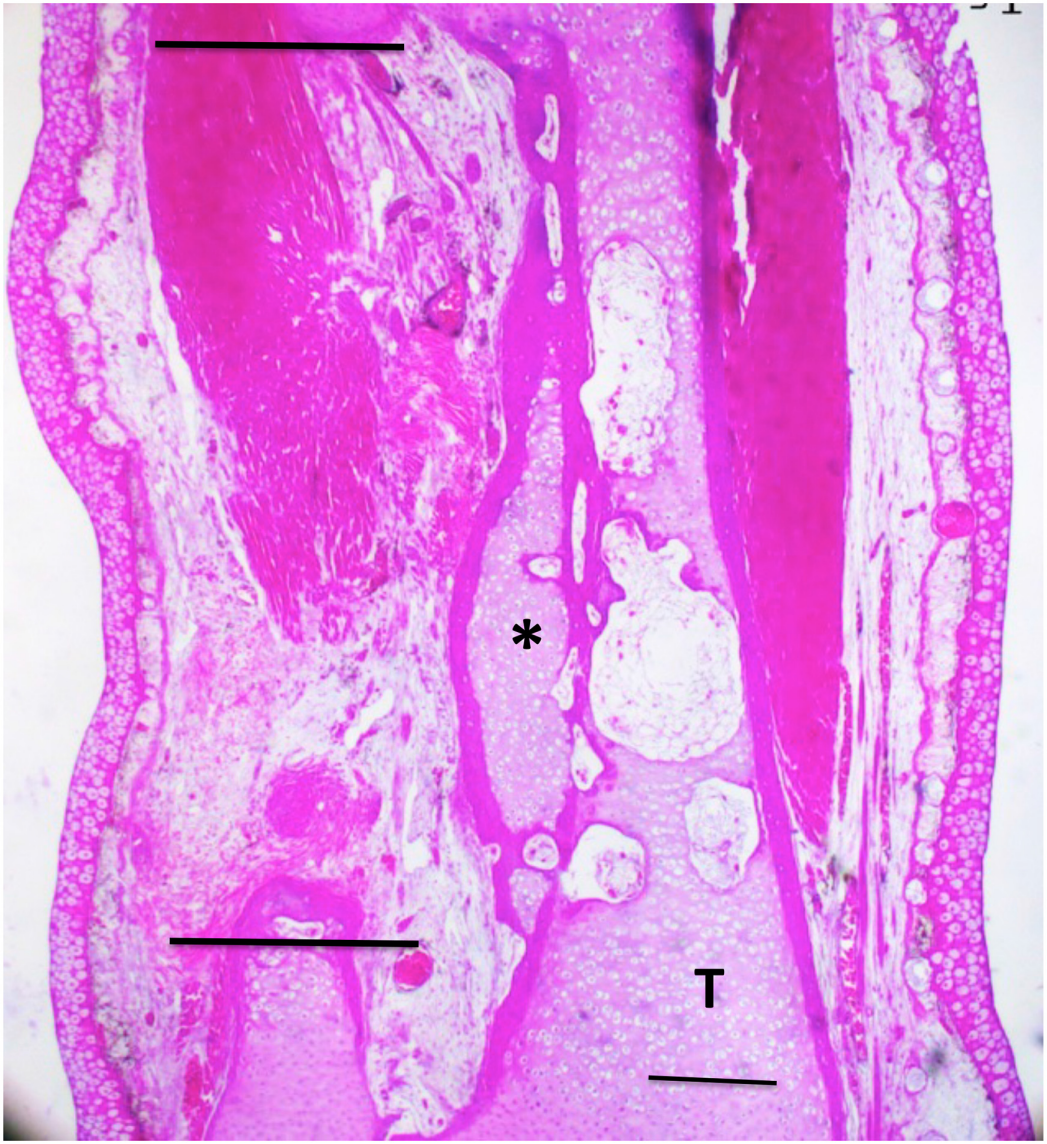
A 50% defect treated with BMP4/HGF, three months post-operation. An irregular bar of cartilage (asterisk) was induced along the proximodistal axis of the tibia (T). Horizontal lines indicate the boundaries of the gap in the fibula. Scale bar = 400 μm.

**Fig 12:**
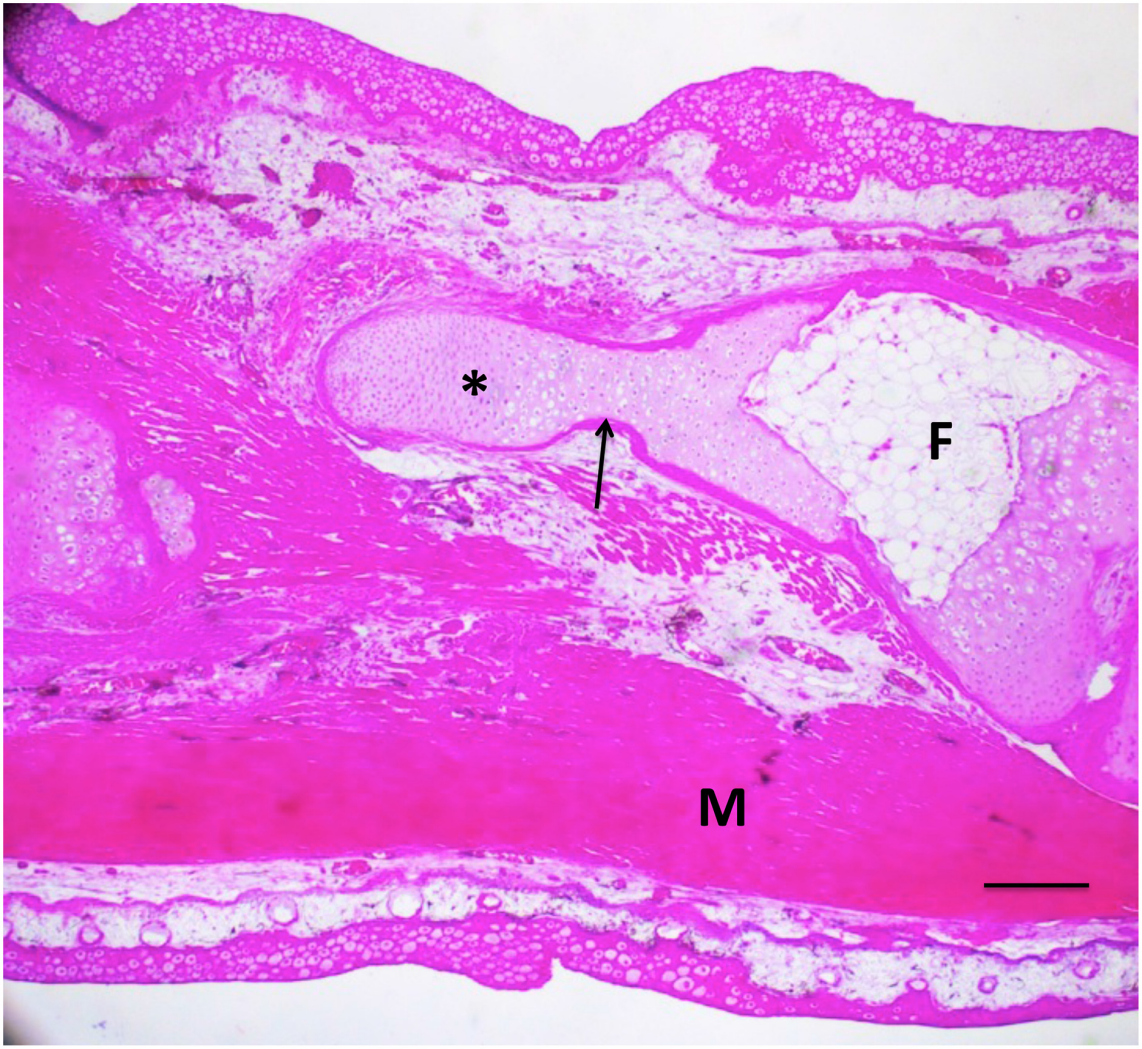
A 50% defect three months after treatment with BMP4/HGF, in which cartilage (asterisk) regenerated proximally from the distal end of the fibula (F) across half the defect. A thin shell of periosteal bone (arrow) covers the regenerated cartilage. M = muscle. Scale bar = 400 μm.

The BMP-4/HGF combination yielded two cases of partial regeneration (Fig 10) and four cases of extensive regeneration out of 20 limbs (Figs 11 and 12). The case illustrated in Fig 11 shows that regeneration of new cartilage surrounded by a shell of bone took place over the length of the gap. The regenerated skeletal tissue was not within the gap, however, but was attached to the side of the tibia, suggesting that its origin was the periosteum of the tibia. Fig 12 shows a case where cartilage regenerated across half the defect from the proximal end of the fibula.

**Fig 13:**
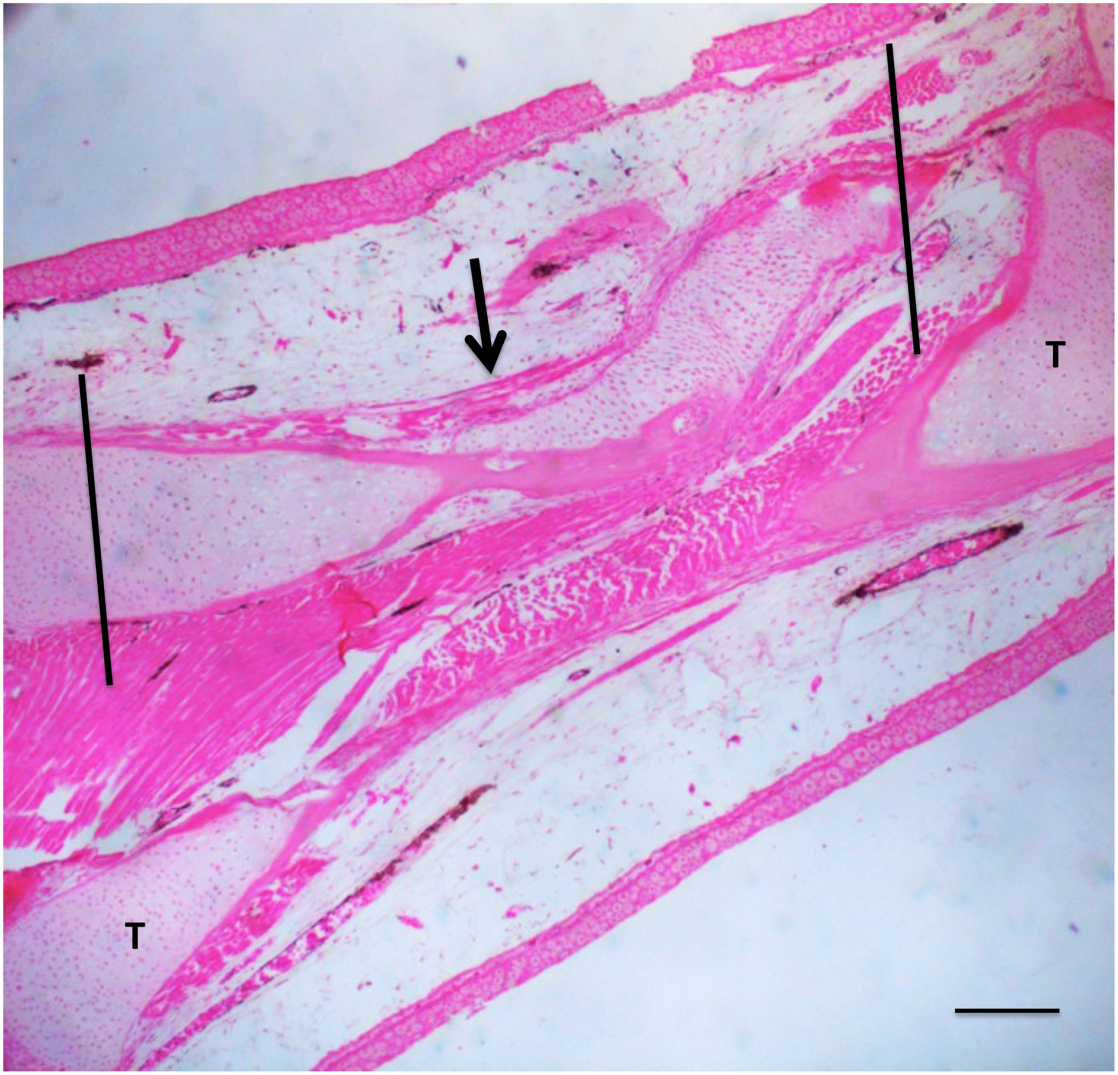
A 50% defect three months after treatment with BMP-4/HGF. Cartilage (arrow) has regenerated across the defect space. Proximal is to the right; distal to the left. Vertical lines indicate approximate proximodistal boundaries of the defect space. T = tibia. Scale bar = 400 μm.

**Fig 14:**
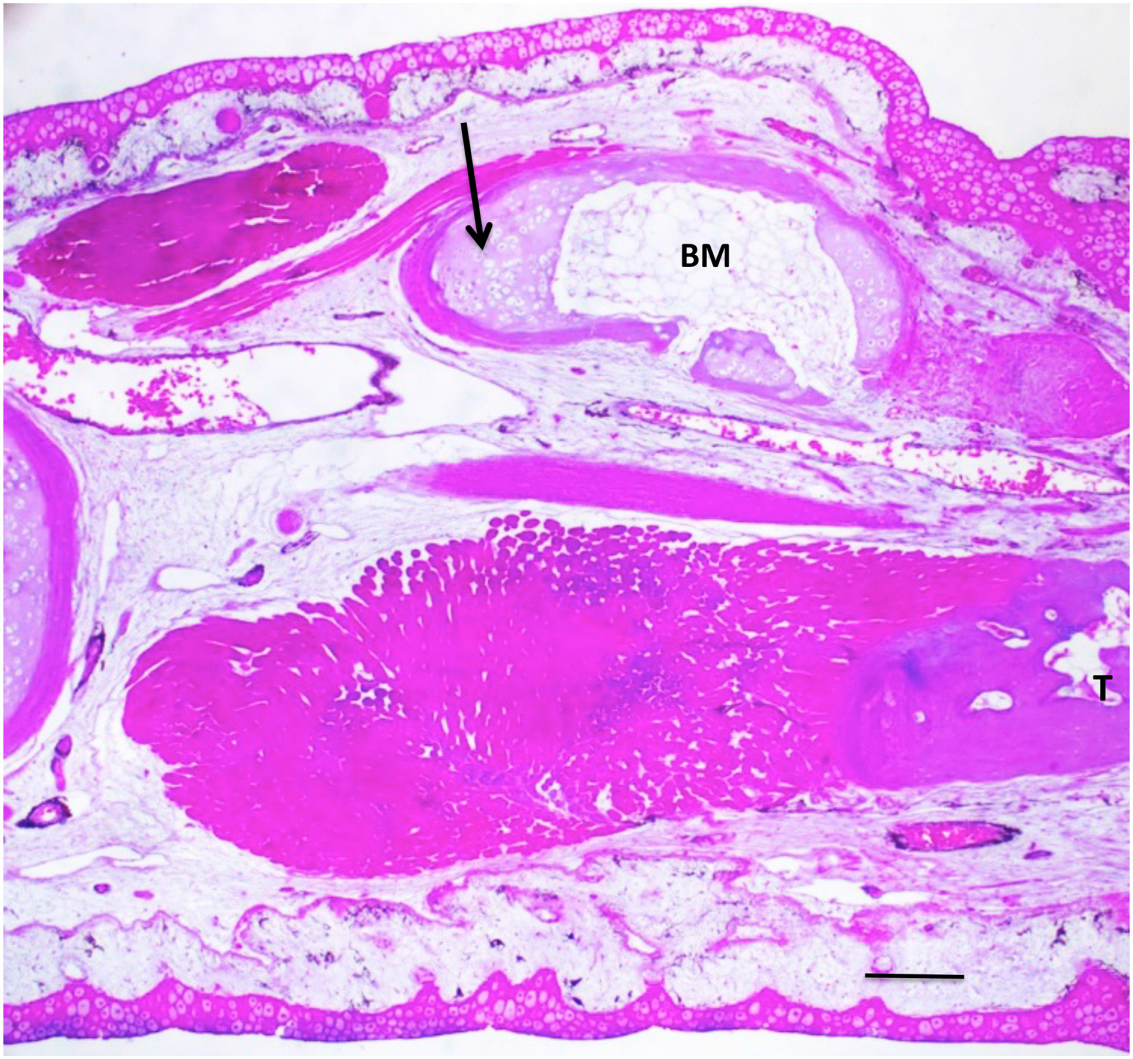
A 50% defect three months after treatment with tissue extract. Cartilage and bone regenerated over nearly the complete length of the defect (arrow). Bone marrow (BM) has formed in the middle of the regenerated bone. T = tibia. Scale bar = 400 μm.

**Fig 15:**
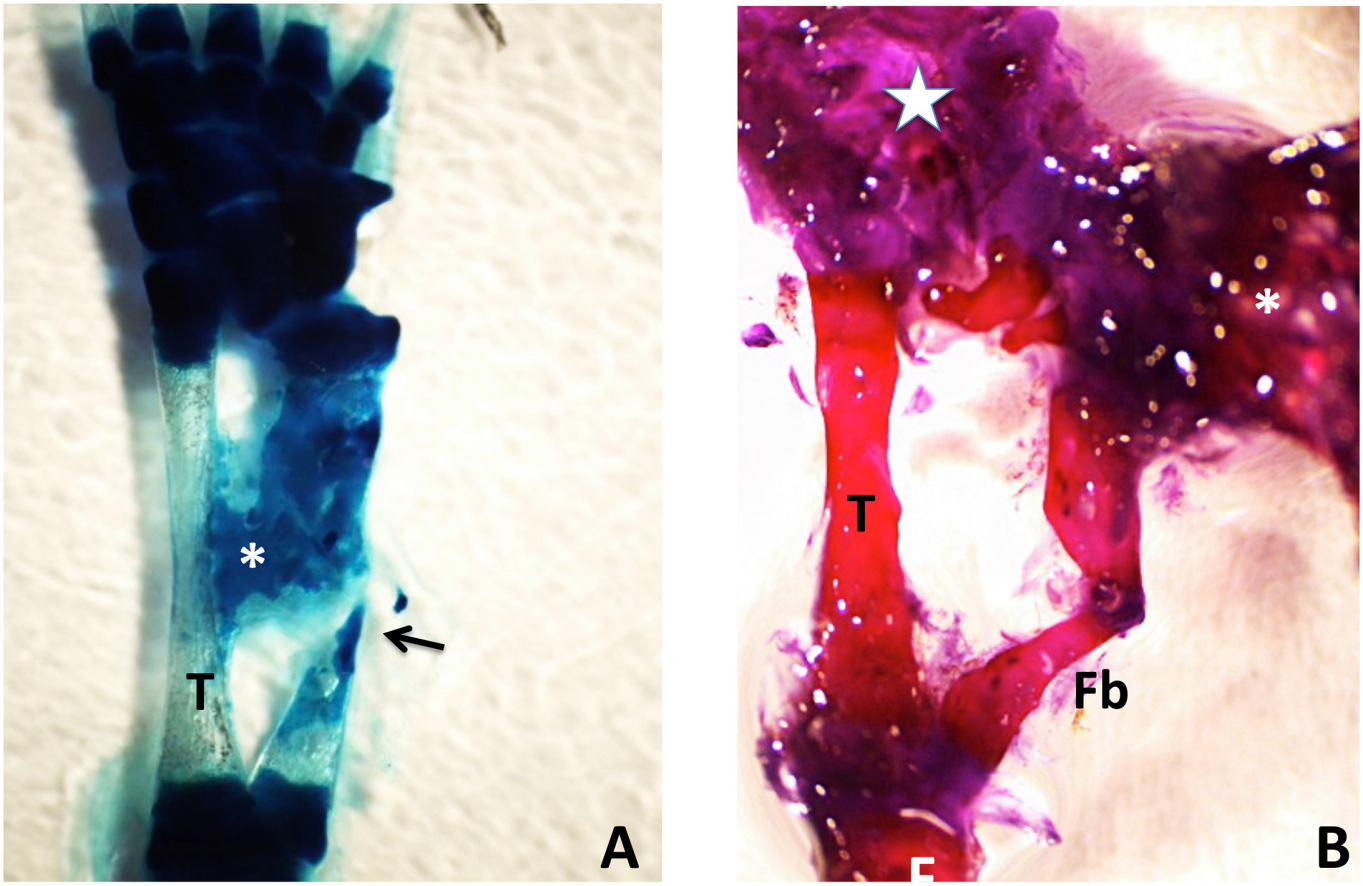
(A) Methylene blue-stained whole mount of a 50% defect three months after treatment with tissue extract. The gap has been completely bridged by an irregular mass of cartilage, which appears to have regenerated primarily from the distal end of the fibula, with only a sliver regenerating from the proximal end (arrow). A cartilage bridge (asterisk), most likely derived from the tibial periosteum, connects the regenerating fibula to the tibia (T). (B) Methylene blue/alizarin red-stained whole mount of a 50% defect three months after treatment with tissue extract. The ends of the fibula were angled with respect to one another so that regeneration from the proximal and distal ends produced a V shape. A supernumerary foot (asterisk) regenerated perpendicular to the fibula. The star indicates the normal foot. Distal is toward the top; proximal is toward the bottom. F = femur; T = tibia; Fb = fibula.

**Fig 16:**
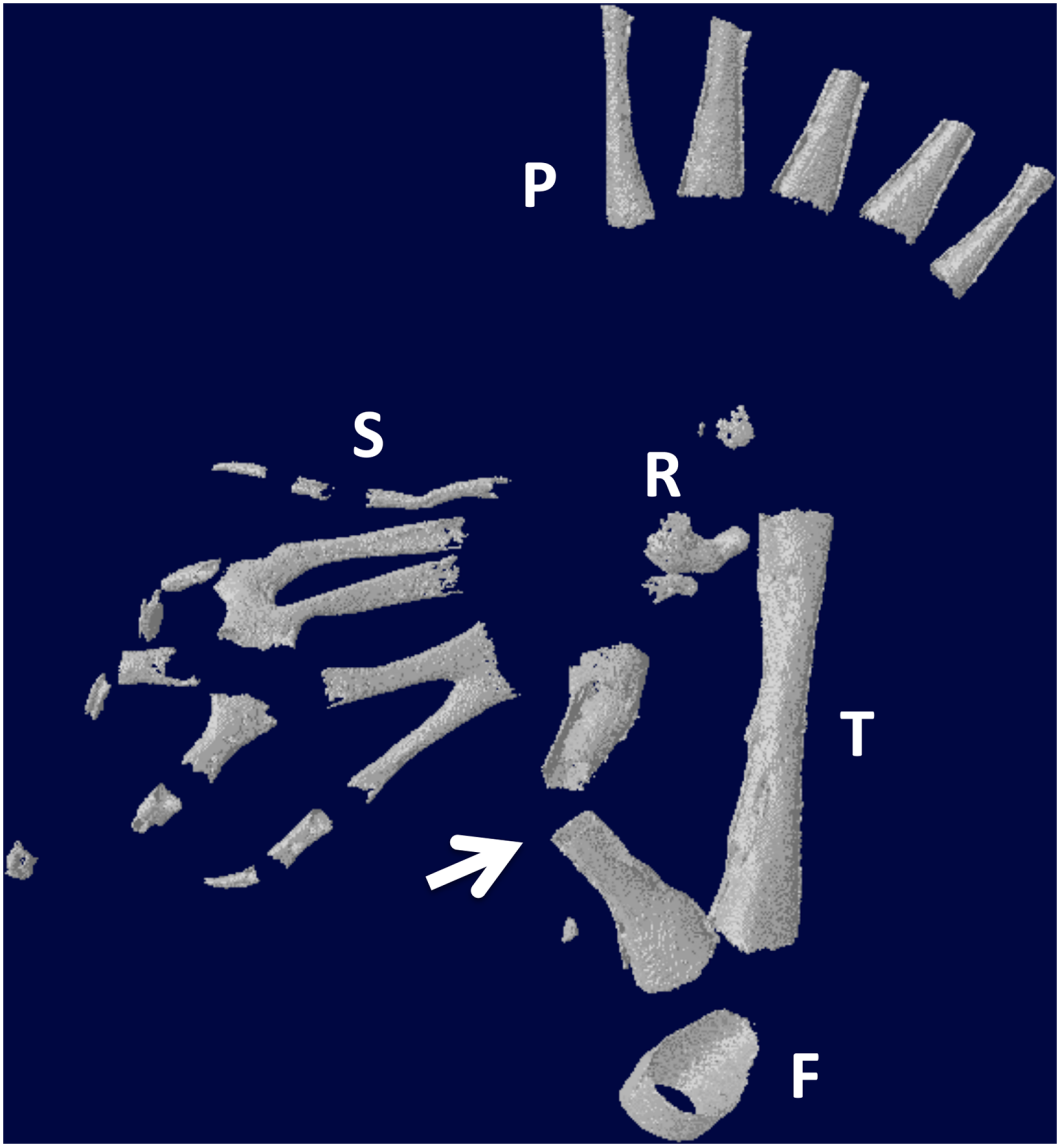
Micro-CT scan of specimen in Fig 15B. The arrow points to the regenerated fibula. S = supernumerary foot skeletal elements; P = skeletal elements of the primary foot. F = femur; T = tibia. R = remnant of distal cut end of the fibula, which has regressed extensively.

Whole limb tissue extract also was effective at inducing regeneration. Of 18 limbs, two had partial regeneration and four had significant regeneration. Figs 13 and 14 show longitudinal sections of limbs in which new cartilage and bone was regenerated over nearly the complete length of the defect. Fig 15 illustrates two other cases treated with tissue extract. In one case, an irregular mass of cartilage has filled the defect and is joined to the tibia by a bridge of cartilage. In the second case, the fibula was regenerated from both distal and proximal stumps. The stumps were angled, so that the regenerated bone formed a shallow V. This specimen had also regenerated a supernumerary foot posteriorly that shared the fibula with the primary foot. The skeletal structures of this specimen are more clearly seen in the micro-CT scan shown in Fig 16.

None of the other growth factor combinations, or blastema protein extract, evoked any regeneration by three months post-implantation.

### BMP-4 Release Kinetics

**Fig 17:**
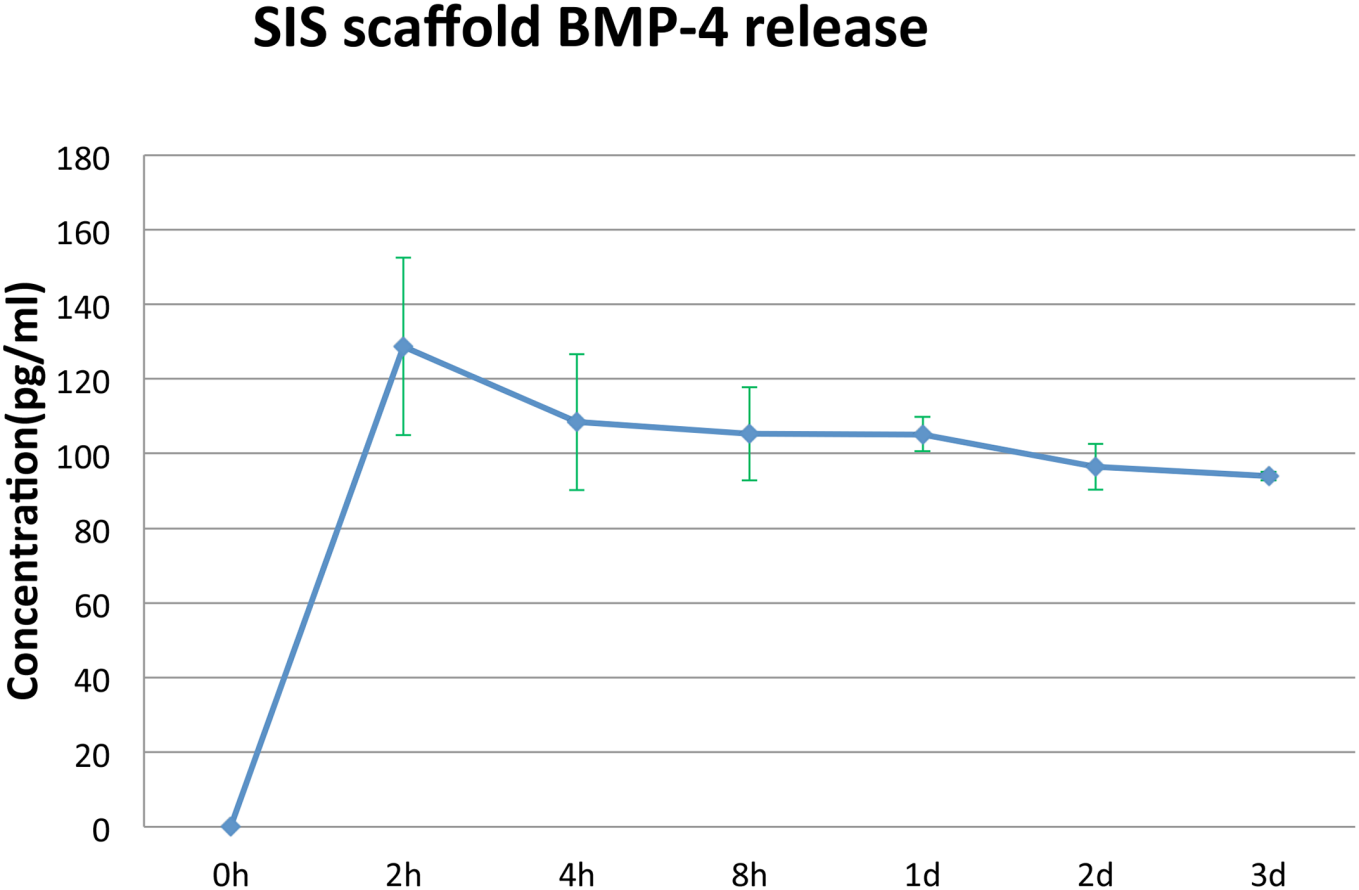
Release kinetics of BMP4 from 8-braid SIS scaffold. Three samples were measured per time point. Bars = SD.

Fig 17 shows the pattern of release kinetics for BMP-4 from the 8-braid SIS scaffold. There was an initial burst of BMP-4 release at 2 hr that declined to 85% of the peak value by 4 hr and then slowly to 77% of the peak value by three days.

## Discussion

### Control segment defects

More than half of the 10% and 20% segment defects showed signs of regeneration two months after operation and 80%-88% of these defects were bridged by three months post-implant as evidenced by micro CT, whole mount staining for cartilage and bone, and histological examination of stained tissue sections. Cartilage developed first and was replaced by bone. Regeneration appeared to take place by extension from one or both ends of the cut bone. Extension from one end continued to the other end, whereas extensions from both ends met in the middle to bridge the gap. These extensions were often at first irregular in appearance but eventually became more regular as endochondral ossification replaced the cartilage template. The impression from histological examination was that the cells providing the chondrocytes for regeneration came from the periosteum at the cut ends of the bone. The bone volume in the middle of the newly regenerated bone of the 10% and 20% segment defects was more than 50% of the total regenerated bone volume.

In contrast to the 10–20% segment defects, the cut ends of the fibula in 40% and 50% defects were capped off and no regeneration took place across the gaps. These defects were filled in by regenerated muscle and connective tissue that lacked organization. The regenerated bone volume was very low in the capped off ends of the 40% and 50% segment defects. Fluorochrome staining showed clearly that regeneration of calcified skeletal tissue took place in the 10% defects within three weeks, whereas none was initiated in the 50% defects. We also observed that the cut ends of the bone in 40% and 50% defects often underwent regression such that the defect space became larger than that created by removing the bone segment. It is possible that such regression sufficient to cause regenerative failure also occurred in the small number of 10% and 20% control defects that did not regenerate by two months. Hutchison et al [21] and Satoh et al [22] observed skeletal regression at the ends of large segment defects in the long bones of young axolotl larvae. They found that neither untreated (control) fibula nor radius regenerated across 25%-54% segment defects (our measurements as derived from their published photographs). If the CSD is defined as the smallest gap at which regeneration fails to occur, their data and ours suggest that the CSD for the fibula of young adult axolotls is greater than 20%, but less than 40%, perhaps as low as 25%. A more detailed study on regeneration across segment defects of 25%, 30%, and 35% will be required to provide a more accurate estimate of the CSD.

### Implants of scaffold alone

SIS scaffold alone did not induce skeletal regeneration across 50% defects. Histological examination indicated that the scaffold degraded over time while the defect was again filled by disorganized muscle fibers and connective tissue. By contrast, Suckow et al [36] found that SIS by itself could promote regeneration across a 30–40% segment defect (as measured from their published photographs) in the radius of adult rats. Cartilage was observed as early as three weeks and bone as early as six weeks. By 6 months, 75% of the defect in all the rats was filled with radiopaque tissue. The difference between their results and ours may be that the SIS in their experiments was prepared *de novo* without the extensive processing done to create the version of SIS that we used and thus may have retained significant amounts of growth factors, such as TGF-β, FGF-2, and VEGF [37] that promoted regeneration.

### Implants of scaffold soaked in growth factors or tissue extracts

We tested several combinations of growth factors all of which contained BMP-4. The only combination that promoted significant regeneration of cartilage and bone across 50% segment defects with any consistency was BMP-4/HGF. Partial regeneration was stimulated in one case of the 7-factor cocktail and two cases of the BMP-4/VEGF combination, but none of the other combinations stimulated regeneration. Consistent with this result, Shi et al [38–40]] found that the effects of combinations of transgenes for IGF-I, BMP-2 and -7, TGF-β1, and FGF-2 on gene expression in cultured articular chondrocytes were not predictable from the regulatory actions of the individual transgenes and thus must be determined empirically.

The ability of BMP-4/HGF to support regeneration in 50% segment defects is not surprising. BMPs are known to be expressed early in axolotl limb regeneration [41–43] and to stimulate the osteogenic competence of mesenchymal stem cells in the periosteum of remodeling and fractured mammalian bone [44–47]. Our results are consistent with reports that BMPs can by themselves elicit the formation of ectopic bone when injected locally into muscle or connective tissue and to initiate the process of new bone formation [33, 48–50]. Most growth factors used to stimulate regeneration over segment defects have used supra-physiological doses (33). There is a considerable amount of work yet to be done to determine the precise number and physiological concentrations of growth factors involved in fracture and segment defect repair, and their temporal pattern of release from ECM and cellular synthesis after injury. Our release kinetics profile for BMP-4 indicates that BMP-4 concentration does not fall off rapidly; after a peak burst at 2 hr and a subsequent 15% decrease by 4 hr, the amount of BMP-4 released is sustained at a relatively steady level of about 75–77% of the peak value over three days. The involvement of HGF would be through its ability to induce expression of BMP receptors [28]. HGF by itself has been found to have an anti-scarring effect in injured laryngeal folds of rats [51] and has long been known to be the principal growth factor that activates satellite cells in muscle regeneration [52]. In addition, HGF inhibits BMP-2-induced generation of osteoblasts in multiple myeloma, prompting the continued proliferation of MSCs [53] and thus could promote chondroblast proliferation and cartilage formation in conjunction with BMP-4.

Why we did not see cartilage and bone induced in all the combinations that contained BMP-4 and HGF is not known. The ratios of growth factor concentrations in combinations other than BMP-4/HGF might have been suboptimal, there may have been functional interference in these combinations that canceled out positive effects, or the growth factors needed to be released in a temporal sequence to be effective. We can also ask why the incidence of regeneration was not higher. One possibility is that there may be obligatory pre-regeneration events that take place in the interval between bone removal and initiation of the regenerative response by growth factors. Even tough BMP-4 release remained high for at least three days in our *in vitro* release kinetics assay, *in vivo* the growth factors migth have suffered substantial inactivation thus reducing their effectiveness to promote regeneration. Since clinical use ultimately demands a one-step surgical protocol that simultaneously removes damaged bone and implants regeneration-promoting factors, research directed design and testing of slow release or timed release scaffolds is of prime importance.

In the treated 50% defects, one case (Fig 11) was noted in which skeletal tissue appeared to arise either totally or in part from the periosteum of the tibia, forming a rod parallel and attached to the tibia (Fig 11). The most likely explanation for this configuration is an extensive unraveling of the scaffold fibers, and/or movement of the scaffold out of the segment defect space toward the tibia, allowing a wider diffusion of the growth factors or extract. In a second case (Fig 15A) skeletal tissue appeared to be regenerated from the fibula, but at the same time a bridge was formed between this tissue and the tibia that appeared partly derived from the tibial periosteum. These results point to the need to develop a scaffold that will release regeneration promoting factors within a confined space having the diameter of the fibula in a temporally sequential fashion.

In BMP-4/HGF-treated 50% defects, as in untreated 10% and 20% defects, the regenerating cartilage appears to grow from either or both cut ends of the fibula, suggesting either a periosteal or cartilage origin. Cartilage contributes only a small percentage of the blastema cells that regenerate cartilage in an amputated axolotl limb, most regenerated chondrocytes arising by the transdifferentiation of dermal fibroblasts [54]. In mammals, the cartilage that repairs fractured long bones arises from mesenchymal stem cells (MSCs) in the periosteum and endosteum [55]. Thus periosteal/endosteal MSCs are the most likely source of the regenerated cartilage in our experiments, but transdifferentiation of dermal or muscle fibroblasts to chondrocytes cannot be ruled out. Labeling studies will be required to trace the actual origin(s) of the regenerating cartilage. The cartilage formation induced by BMP-4/HGF is succeeded by ossification to complete the regenerative process through pathways triggered by factors such as VEGF that are expressed by hypertrophied chondrocytes [56].

Protein extract of whole limb tissues was a somewhat more effective promoter of skeletal tissue regeneration than BMP-4/HGF across 50% gaps in the fibula, whereas protein extract of regeneration blastemas had no effect. This result suggests that limb tissue extract contains factors in addition to BMPs and HGF, or simply more optimum concentrations of BMP-s and HGF that initiate the molecular cascade leading to cartilage regeneration and ossification. Pekarinen et al [57] and Tolli [58] reported similar results with reindeer bone extract alone on CSDs of rabbit radius and rat femur, respectively. It would be instructive to investigate protein extracts of individual limb tissues for their effects on CSD regeneration, and to conduct targeted quantitative proteomic analyses of extracts to identify the effective factors. Why protein extract of regeneration blastemas failed to induce regeneration across segment defects is unknown, but one explanation could be that the factors present in blastemal extract are not temporally matched to what is required after removal of a bone segment, whereas such factors would be available in the extract of intact limb tissue, particularly factors sequestered in bone matrix.

Feng et al [19] demonstrated a failure to bridge large segment defects made in one of the two tarsal bones of the unamputated adult *Xenopus* hind limb. They used a biocompatible 1,6 hexanedioldiacrylate (HDDA) scaffold loaded with BMP-4 and VEGF to induce bridging of the CSD by a cartilage template, which was followed by the beginning of osteogenesis in the mid-region of the cartilage. The scaffold did not act as an osteoinductive substrate, but rather to deliver the growth factors over the whole length of the CSD, and was pushed to one side of the regenerating cartilage. Untreated CSDs formed only fibrous scar tissue. A disadvantage of this model was that the HDDA scaffold was not biodegradable.

Satoh et al [22] found that microbeads loaded with BMP-2 were able to stimulate cartilage regeneration across segment defects of various lengths made in the radius of small (3 cm) axolotl larvae. They speculated that the regenerated cartilage was derived from fibroblasts that differentiated directly into chondrocytes without first dedifferentiating, since they proliferated without expressing *Prrx-1*. *Prrx-1* is a transcription factor that plays a role in limb development and fibroblast migration [59, 60] and is a marker for the dedifferentiated cells that form the regeneration blastema in an amputated axolotl limb. Blastema cells grafted into a segment defect of the radius also differentiated into cartilage. Furthermore, removing the skin over a defect to create a wound epidermis and deviating a nerve into the CSD, two conditions associated with amputation-induced limb regeneration, resulted in cartilage regeneration by the proliferation of fibroblasts that did express *Prrx-1*. This result suggested that the nerve and wound epidermis provided signals that induced histolysis and dedifferentiation to produce blastema cells, similar to what happens in the amputated limb.

In our experiments, wound epidermis may have been formed by sufficient injury to the limb to induce the regeneration of a supernumerary foot in one case (Figs 15 and 16). The supernumerary foot was composed only of digits and possibly tarsals, and arose from the region of the distal remnant of the fibula and the distal part of the regenerated fibula. This brings up the possibility that regeneration of the distal fibula and the supernumerary foot was coupled through the formation of a blastema, the growth of which requires a signaling circuit between nerves and wound epidermis [61–63]. It would be of great interest to explore the potential role of nerves in stimulating regeneration in segment defects.

## Conclusion

We have established that the axolotl (and most likely other urodele species) can serve as an inexpensive and surgically amenable model to screen different combinations of factors for their ability to promote regeneration of cartilage and bone across a CSD. The model has established that a combination of BMP-4 and HGF, as well as whole limb issue extract is effective in evoking regeneration across gaps of 50% or greater in the axolotl fibula. This model will likely also be amenable to the screening of different scaffold types to optimize the osteoconductive and osteoinductive characteristics of biomaterials and architectures for delivery of cartilage cascade-initiating and sustaining factors that promote cartilage regeneration across defects of critical size or greater in long bones. The model may also be informative about bone regeneration under conditions of weightlessness, such as are encountered on long space flights, since limb and skeletal regeneration in axolotls takes place in a buoyant environment that mimics reduced gravity.
